# Single nucleotide polymorphisms in microRNA genes are associated with cervical cancer susceptibility in a population from Xinjiang Uygur

**DOI:** 10.18632/oncotarget.12212

**Published:** 2016-09-23

**Authors:** Jie Yang, Zegao Zhang, Wen Guo, Yuhua Ma, Raila Muhammed Emin, Karima Abudubari, Glmira Hayrat, Hasiyet Wali, Xiaoli Qi, Chunhua Liu, Miaomiao Ma, Pulat Nurbek

**Affiliations:** ^1^ Radiotherapy Second Department, People's Hospital of Xinjiang Uyghur Autonomous Region, Urumqi, Xinjiang, China; ^2^ Inner Mongolia Medical University, Hohhot, Inner Mongolia Autonomous Region, China

**Keywords:** association study, cervical cancer, microRNA gene, single nucleotide polymorphisms (SNPs)

## Abstract

The goal of this study was to explore the correlation between single nucleotide polymorphisms (SNPs) and susceptibility to cervical cancer (CC) in a population from Xinjiang Uygur. Participating were 247 patients with CC and 285 healthy women. Fourteen SNPs in nine miRNA genes were selected. Odds ratios (ORs) and 95% confidence intervals (95% CIs) were calculated using unconditional logistic regression analysis. Multivariate logistic regression analysis was used to assess the correlation of SNPs with CC. The minor allele “C” of rs300574 in *SPRY1* was associated with an increased risk of CC based on analysis of the allele, codominant, recessive and log-additive models, but an opposite result was found with the over-dominant model. The minor allele “C” of rs1042725 in *HMGA2* was associated with an increased risk of CC in the allele, dominant and log-additive models. In clinical stage III/IVCC patients, rs4728 in *SPRY2* was associated with decreased risk. Finally, rs3744935 in *BCL2* was associated with CC in the allele and codominant models. In sum, we have detected associations between four SNPs, rs300574 (*SPRY1*), rs3744935 (*BCL2*), rs1042725 (*HMGA2*), and rs4728 (*SPRY2*), and CC risk in women from Xinjiang Uygur.

## INTRODUCTION

Cervical cancer (CC) is one of the most common malignancies among women worldwide, particularly in developing countries [1].CC accounts for 9% of the total new cancer cases and 8% of the total cancer deaths among women [2]. Despite preventive strategies and innovative treatments, it is estimated that by the year 2020, there will be 609,270 new CC cases and 317,727 deaths [1]. Although experimental and epidemiological evidence indicates that infection with high-risk human papillomavirus (hrHPV) is the main CC etiologic agent, it is not sufficient to cause the malignancy [3]. Rather, CC results from interactions of various factors, including the HPV infection, environmental, behavioral, and genetic factors [4, 5].

Pathogenesis of CC is a multistep process that results from the accumulation of several genomic alterations, and is characterized by unrestricted proliferation, invasion and metastasis [6]. MicroRNAs (miRNAs) are small (18–25 nucleotides) non-coding RNAs that modulate post-transcriptional mRNA expression. Since miRNAs regulate expression of genes involved in cell proliferation, differentiation and apoptosis, they can function as potential oncogenes or tumor suppressors [7–11]. We have previously found that chromosome mutations and the change of single nucleotide polymorphisms (SNPs) are important factors that induce malignant transformation of cervical epithelial cells [12].

In this case-control study, we have investigated the relationship between the SNPs in miRNA genes and the risk of CC, and performed a comprehensive association analysis in China Xinjiang Uygur population.

## RESULTS

A total of 247 CC patients and 285 healthy subjects were enrolled in our study. Detailed information about the SNPs selected is presented in Table 1. As a risk factor, the minor allele of each SNP was compared with the wild-type allele. All of the tested SNPs were in agreement with the HWE in the control population of this study (*p* > 0.05) except for rs8756 (*p* = 0.040) and rs11175982 (*p* = 0.024); therefore, they were excluded from the analysis. Comparing the differences in frequency distributions of alleles between cases and controls by χ^2^ test, we found there is a correlation between two loci (rs300574, *SPRY1*, OR = 1.312, 95% CI: 1.034–1.677, *p* = 0.026; rs1042725, *HMGA2*, OR = 1.309, 95% CI: 1.009–1.699, *p* = 0.043) and increased CC development under allele model. On the contrary, the T allele of rs3744935 (*BCL2*, OR = 0.450, 95% CI: 0.214–0.947, *p* = 0.031) was found to be a protective factor (Table 1). Besides, the other loci under allele model had not been found to be associated with the disease. We also performed a Bonferroni correction and determined that none of the SNPs showed statistically significant associations with CC risk.

**Table 1:** Basic information of SNPs in this study

SNPs	MiRNA	Gene	Chr	Band	Role	Alleles	MAF		HWE	OR	95% CI	*p*-x^2^
						A/B	control	case	*p*-value			
rs2431	hsa-let-7b	*FNDC3B*	3	3q26.31	3*2032* UTR	G/A	0.396	0.421	0.387	1.106	0.865–1.413	0.423
rs300574	hsa-miR-21	*SPRY1*	4	4q28.1	3*2032* UTR	C/T	0.446	0.514	0.15	1.312	1.034–1.677	**0.026**
rs4272	hsa-miR-21	*CDK6*	7	7q21.2	3*2032* UTR	G/A	0.105	0.105	0.104	1.000	0.675–1.481	1.000
rs1042725	hsa-let-7b	*HMGA2*	12	12q14.3	3*2032* UTR	C/T	0.282	0.340	0.663	1.309	1.009–1.699	**0.043**
rs8756	hsa-let-7b	*HMGA2*	12	12q14.3	3*2032* UTR	C/A	0.225	0.237	0.04	1.072	0.805–1.426	0.635
rs11175982	hsa-let-7b	*HMGA2*	12	12q14.3	3*2032* UTR	C/T	0.479	0.510	0.024	1.134	0.890–1.443	0.309
rs4728	hsa-miR-21	*SPRY*2	13	13q31.1	3*2032* UTR	C/A	0.260	0.310	1	1.279	0.979–1.671	0.071
rs11911	hsa-miR-21	*SPRY2*	13	13q31.1	3*2032* UTR	C/A	0.414	0.398	1	0.936	0.732–1.196	0.595
rs12942088	hsa-mir-133b	*CD68*	17	17p13.1	Promoter	C/T	0.486	0.540	0.708	1.126	0.880–1.440	0.343
rs9901673	hsa-mir-133b	*CD68*	17	17p13.1	Coding exon	A/C	0.382	0.411	0.355	1.114	0.800–1.552	0.522
rs3744935	hsa-miR-21	*BCL2*	18	18q21.33	3*2032* UTR	T/C	0.150	0.164	1	0.450	0.214–0.947	**0.031**
rs7529	hsa-let-7b	*GZMM*	19	19p13.3	Promoter	T/A	0.135	0.142	0.318	1.057	0.746–1.498	0.755
rs8708	hsa-miR-21	*JAG1*	20	20p12.2	3*2032* UTR	C/T	0.335	0.326	0.69	0.959	0.743–1.240	0.751
rs7828	hsa-miR-21	*JAG1*	20	20p12.2	3*2032* UTR	C/A	0.174	0.186	0.407	1.089	0.796–1.490	0.595

SNPs: Single nucleotide polymorphisms; MiRNA: microRNA; Chr: chromosome; MAF: Minor allele frequency; HWE: Hardy-Weinberg equilibrium; OR: Odds ratio; CI: Confidence interval.

*P-*value was calculated by Pearson's χ^2^ test.

*P* value < 0.05 indicates statistical significance.

Further model analysis was conducted by unconditional logistic regression analysis and only the SNPs associated with CC were included (Table 2). We found that rs300574 was associated with CC under codominant model (OR = 1.79, 95% CI: 1.10–2.91, *p* = 0.004), recessive model (OR = 1.97, 95% CI: 1.31–2.97, *p* = 0.001), over-dominant model (OR = 0.66, 95% CI: 0.47–0.93, *p* = 0.018) and log-additive model (OR = 1.31, 95% CI: 1.03–1.67, *p* = 0.026). We also noticed that rs1042725 was associated with increased CC risk in dominant model (OR = 1.50, 95% CI: 1.06–2.11, *p* = 0.021) and log-additive model (OR = 1.34, 95% CI: 1.02–1.76, *p* = 0.035). Besides, rs3744935 actually decreased the risk of CC under codominant model (OR = 0.44, 95% CI: 0.21–0.93, *p* = 0.026). In addition, no positive results were observed after Bonferroni correction.

**Table 2:** Unconditional logistic regression analysis of the association between the single-nucleotide polymorphisms and CC risk

SNPs	Model	Genotype	Controls (*n* %)	Cases (*n* %)	OR (95% CI)	*P*-value	AIC	BIC
rs300574	Codominant	T/T	81 (28.4%)	66 (26.7%)	1			
		T/C	154 (54%)	108 (43.7%)	0.86 (0.57–1.29)			
		C/C	50 (17.5%)	73 (29.6%)	1.79 (1.10–2.91)	**0.004**	729.5	742.4
	Dominant	T/T	81 (28.4%)	66 (26.7%)	1			
		T/C-C/C	204 (71.6%)	181 (73.3%)	1.09 (0.74–1.59)	0.660	738.6	747.2
	Recessive	T/T-T/C	235 (82.5%)	174 (70.5%)	1			
		C/C	50 (17.5%)	73 (29.6%)	1.97 (1.31–2.97)	**0.001**	728.1	736.6
	Over-dominant	T/T-C/C	131 (46%)	139 (56.3%)	1			
		T/C	154 (54%)	108 (43.7%)	0.66 (0.47–0.93)	**0.018**	733.2	741.7
	Log-additive	---	---	---	1.31 (1.03–1.67)	**0.026**	733.9	742.4
rs1042725	Codominant	T/T	145 (50.9%)	101 (40.9%)	1			
		C/T	119 (41.8%)	124 (50.2%)	1.50 (1.05–2.14)	0.070	735.5	748.3
		C/C	21 (7.4%)	22 (8.9%)	1.50 (0.79–2.88)			
	Dominant	T/T	145 (50.9%)	101 (40.9%)	1			
		C/T-C/C	140 (49.1%)	146 (59.1%)	1.50 (1.06–2.11)	**0.021**	733.5	742
	Recessive	T/T-C/T	264 (92.6%)	225 (91.1%)	1			
		C/C	21 (7.4%)	22 (8.9%)	1.23 (0.66–2.29)	0.520	738.4	746.9
	Over-dominant	T/T-C/C	166 (58.2%)	123 (49.8%)	1			
		C/T	119 (41.8%)	124 (50.2%)	1.41 (1.00–1.98)	0.051	735	743.5
		---	---	---	1.34 (1.02–1.76)	**0.035**	734.4	742.9
rs3744935	Codominant	C/C	260 (91.2%)	237 (96%)	1			
		T/C	25 (8.8%)	10 (4%)	0.44 (0.21–0.93)	0**.026**	733.8	742.4
		T/T	0	0	---	---	---	---

SNPs: Single nucleotide polymorphisms; OR: Odds ratio; CI: Confidence interval.

*P*-value was calculated by Pearson's χ^2^ test.

*P*-value < 0.05 indicates statistical significance.

The association between SNPs and different CC patient clinical subtypes was also analyzed only positive results (Table 3). From the results, we found that rs300574 (OR = 1.404, 95% CI: 1.060–1.860, *p* = 0.018) and rs1042725 (OR = 1.403, 95% CI: 1.040–1.892, *p* = 0.026)were associated with clinical stage I/IICC patients. Rs4728 (OR = 1.473, 95% CI: 1.029–2.110, *p* = 0.034) was associated with an increased CC risk in clinical stage III/IVcases. In addition, rs1042725 (OR = 3.266, 95% CI: 1.196–8.918, *p* = 0.015) was associated with adenomatous carcinoma patients. Rs300574 (OR = 1.330, 95% 1.042–1.698, *p* = 0.022) and rs3744935 (OR=0.371, 95% CI: 0.166–0.831, *p* = 0.012)were associated with squamous carcinoma patients.

**Table 3:** The association between SNPs and different clinical subtypes of CC patients

Variables	rs300574			rs1042725			rs4728			rs3744935		
	OR	95% CI	*P*	OR	95% CI	*P*	OR	95% CI	*P*	OR	95% CI	*P*
Clinical stage												
I/II	1.404	1.060–1.860	**0.018**	1.403	1.040–1.892	**0.026**	1.176	0.860–1.606	0.309	0.524	0.224–1.227	0.13
III/IV	1.244	0.891–1.738	0.2	1.129	0.785–1.624	0.513	1.473	1.029–2.110	**0.034**	0.365	0.109–1.225	0.089
Pathological types												
adenomatous carcinoma	0.968	0.355–2.634	0.949	3.266	1.196–8.918	**0.015**	0.658	0.185–2.341	0.515	3.114	0.671–14.453	0.127
squamous carcinoma	1.330	1.042–1.698	**0.022**	1.266	0.973–1.648	0.079	1.304	0.996–1.707	0.053	0.371	0.166–0.831	**0.012**

SNPs: Single nucleotide polymorphisms; OR: Odds ratio; CI: Confidence interval.

*P -*value was calculated by Pearson's χ^2^ test.

*P*-value <0.05 indicates statistical significance.

## DISCUSSION

The goal of this study was to explore the correlation of SNPs with the susceptibility to CC in Xinjiang Uygur population. We have identified four SNPs: rs300574 (*SPRY1*), rs3744935 (*BCL2*), rs1042725 (*HMGA2*), and rs4728 (*SPRY2*) that are associated with CC risk in Xinjiang Uygur population.

The minor allele “C” of rs300574 in *SPRY1* gene was associated with an increased risk of CC based on the analytic results of the allele, codominant, recessive and log-additive model, but an opposite result was found in the over-dominant model. The minor allele “C” of rs1042725 in *HMGA2* gene was associated with an increased risk of CC under the allele, dominant and log-additive model. *HMGA2* rs1042725 has been reported to contribute to height variability in European population [13], and US Caucasian and Chinese populations [14], but not in Korean [15] and Japanese population [16] To our knowledge, this is the first study that reports the association between rs1042725 in *HMGA2* gene and cancer. In addition, in clinical stage III/IVCC patients, rs4728 in *SPRY2* gene was associated with a decreased risk. Finally, we also found that the minor allele “T” of rs3744935 in *BCL2* gene was associated with CC under allele and codominant model. The above two loci have not been reported previously.

Recent studies have revealed that miRNA deregulation correlates with various human cancers and is involved in the initiation and progression of human tumors [17]. Since the first miRNA lin-4 was discovered in Caenorhabditis elegans, miRNA-dependent gene regulation has been widely investigated [18, 19]. As miRNAs can inhibit mRNA translation or induce mRNA degradation, thus regulating a wide range of biological processes including cell proliferation, differentiation and apoptosis abnormal miRNA expression is a common feature of human cancers.

Homo sapiens miR-21 (hsa-miR-21) is one of the first miRNAs detected in the human genome and is the major oncogene up-regulated in many types of human cancer including glioblastoma multiforme [20], breast [21], lung [22], esophageal gastrointestinal [23], hepatocellular [24], cholangiocarcinoma [25], pancreatic [26], ovarian [27], bladder [28], NK-cell lymphoma [29], laryngeal carcinoma [30] and tongue squamous cell carcinoma [31]. Aldaz et al. found that by direct 3′ -UTR binding, miR-21 up-regulation decreases *SPRY1* expression, thus contributing to cancer development [32]. Thus, we speculate that the mechanism by which miRNA gene *SPRY1* increases the risk of CC development might be similar to the study by Aldaz et al. *SPRY2* has also been reported to promote apoptosis of cancer cells which is associated with activation of the phosphatase and tensin homolog deleted on chromosome 10 (PTEN) pathway and the blockade of Ras-Raf-Erk signaling [33]. In addition, it was suggested high *BCL2* expression were associated with unfavourable prognostic in diffuse large B-cell lymphoma [34]. Sung Han Kim et al. found that *BCL2* gene might play distinctive roles in cisplatin resistance in bladder cancer [35].

Hsa-let-7b, a member of hsa-let-7 family of tumor suppressor miRNAs, possesses a high homology to 3′-UTR of transcripts encoding for proteins involved in proliferation, differentiation and cell death [36]. *HMGA2* (High Mobility Group AT-2 hook) belongs to *HMG* (High Mobility Group) family of proteins, and is an essential component of the enhanceosome, which drives DNA to the transcriptional complexes [37, 38]. *HMGA2* expression correlates with metastases and reduced survival, and is increased in several malignancies, such as lung, prostate, colon, pancreatic, gastric and breast cancer [39–43]. In addition, it has been reported that hsa-let-7b is able to regulate targets *HMGA2* and the absence of hsa-let-7b has been linked to high levels of *HMGA2* [39]. Di Fazio et al. found that *HMGA2* expression was controlled by tumor suppressor miRNA hsa-let-7b after inhibition of deacetylases in liver cancer cell lines [44]. Therefore, it seems plausible that downregulation of hsa-let-7b leads to increased levels of *HMGA2*, which further contributes to the generation of liver cancer. Though the association between hsa-let-7b, *HMGA2* and CC development has not been reported previously, we hypothesize that guessed the functional mechanism might be similar to that in liver cancer.

We found no statistically significant association between SNPs and the risk of CC using Bonferroni correction in our statistical analysis. This may be due to the relatively small sample size, the selection criteria for SNPs (minor allele frequency [MAF] > 5%), and the weakness of Bonferroni correction itself (the interpretation of a finding depends on the number of other tests performed). Future studies should confirm our conclusions using a larger sample size, other population groups, consider patients' age, as well as other factors, such as smoking, bacterial and viral infections, and social status.

In summary, we have identified novel associations between four SNPs, rs300574 (*SPRY1*), rs3744935 (*BCL2*), rs1042725 (*HMGA2*) and rs4728 (*SPRY2*) with CC risk in Xinjiang Uygur population. This may provide new strategies for CC screening and identify new genes and mechanisms of CC pathogenesis.

## MATERIALS AND METHODS

### Study participants

In this case-control study, a total of 247 patients with invasive cervical cancer and 285 healthy women were recruited at People's Hospital of Xinjiang Uyghur Autonomous Region from January 2014 to June 2016. The included patients were recently diagnosed by cervical biopsy and histopathologically confirmed as primary CC. We excluded the patients with other cancers who underwent radiotherapy or chemotherapy. The controls who had an annual health check were recruited from the health checkup center of the same hospitals. All the controls were matched with the cases, and all of them had no history of cancer.

Tumors were staged according to International Federation of Gynecology and Obstetrics (FIGO) classification. The factors that could influence the mutation rate were minimized. All participants enlisted were women at least 18 years old with good mental condition and no blood relationship going back three generations. Besides, both cohorts belong to the same ethnically homogenous population (Xinjiang Uygur population).

Informed consents were obtained from all participants and the study protocols were approved by the institutional review board of People's Hospital of Xinjiang Uyghur Autonomous Region.

### SNP selection and genotyping

Validated SNPs, associated with other cancers published in previous studies, were selected with a minor allele frequency (MAF) > 5% in the HapMap Asian population [45–53]. Venous blood samples (5 ml) were collected from each patient during laboratory examination. For patients, blood was collected prior to radiation or chemotherapy. DNA was extracted from whole blood samples using the Gold Mag-Mini Whole Blood Genomic DNA Purification Kit (GoldMag Ltd., Xi'an, China) and stored at−80°C after centrifugation. DNA concentration was evaluated by spectrometry (DU530 UV/VIS spectrophotometer, Beckman Instruments, Fullerton, CA, USA). Sequenom MassARRAY Assay Design 3.0 software (Sequenom, Inc, San Diego, CA, USA) was used to design multiplexed SNP Mass EXTEND assay. The SNP genotypes were performed by a Sequenom MassARRAY RS1000 (Sequenom, Inc) according to the standard protocol recommended by the manufacturer. The Sequenom Typer 4.0 Software™ (Sequenom, Inc) was used to perform data management and analyses. All primers were made by Sangon (Shanghai, China), their sequences are available upon request. The corresponding primers used for each SNP in our study are listed in Table 4. As a result, fourteen SNPs were selected including: rs2431, rs300574, rs4272, rs1042725, rs8756, rs11175982, rs4728, rs11911, rs12942088, rs9901673, rs3744935, rs7529, rs8708 and rs7828. The SNPs genetic information included in this study is shown in Table 1.

**Table 4:** Primers used for this study

SNP_ID	1st-PCRP	2nd-PCRP	UEP_SEQ
rs2431	ACGTTGGATGTTTTAGGT CAGTCTTGTTCC	ACGTTGGATGATGCACTAG ATGCTCTTATC	CAGAAGATTAATAATACT ACAGATG
rs300574	ACGTTGGATGGGGTCAGGG TAAACCATCAT	ACGTTGGATGCAAAAAACA GCCACAACTTG	AAAAAACAGCCACAACT TGAAAGCTA
rs4272	ACGTTGGATGGATAAAA CACCTAGATACCC	ACGTTGGATGCTAAGCCCCC AAATAAGCTG	AAATAAGCTGCATGCATT TGTAACA
rs1042725	ACGTTGGATGGCATTGC TTGTGTATAGCAG	ACGTTGGATGCCCCACTA CTCAATACTACC	ACTACCTCTGAATGTTACAA
rs8756	ACGTTGGATGCTCCTGCAA ACATACCTAAC	ACGTTGGATGTTCTTTGC TGTTGTTGGTCG	gGTTGTTGGTCGCAGCTA
rs11175982	ACGTTGGATGCCTGCTCTT TGTTACAGTTC	ACGTTGGATGATGACTTG TGAGTGTCTCCC	ACTTGTGAGTGTCTCCCT CCTTGATCT
rs4728	ACGTTGGATGGTGGTTCA GTATTATGTACG	ACGTTGGATGAATTGTGTG AACTTGGAAGC	TTGGAAGCACACCAATCT
rs11911	ACGTTGGATGGTTGGTTG CATATGTGTAT	ACGTTGGATGCCCTAGGA ACTTTGAAAAACC	ACAGTAATGAGGATTTTTTCT
rs12942088	ACGTTGGATGTCTAGCCAC CTCTCTCTTAG	ACGTTGGATGACCCATGT GACACTGTTGAC	ACTGTTGACTTTGCC CTGA
rs9901673	ACGTTGGATGCATGGCGGT GGAGTACAATG	ACGTTGGATGCGTCTAGTG GTAGCAATGAG	AAGGGAAGGAGGTT ACTTAC
rs3744935	ACGTTGGATGCCTCTACA GTGATACATGTC	ACGTTGGATGTGTGGCTG GGCCTGTCACC	aGCCCTCCAGGTAGGCCC
rs7529	ACGTTGGATGTGCCTGT CAGCTCCAGGTC	ACGTTGGATGCAAAGCAGG GAAAAGAGTCG	CGGCCACGGTGAATCCGT CCACTC
rs8708	ACGTTGGATGGAGCTTAC GTAGTTCTACCG	ACGTTGGATGTACCCCGATT ACCAGAACAG	AGGGTGGCAGAAGCACA
rs7828	ACGTTGGATGGTGGTTCA GTATTATGTACG	ACGTTGGATGAATTGTGTGAA CTTGGAAGC	TTGGAAGCACACCAATCT

### Statistical analysis

All statistical analyses were conducted by SPSS version 17.0 statistical package (SPSS, Chicago, IL, USA) and Microsoft Excel. Pearson's χ^2^ test was used to compare the distribution of categorical variables and Student's *t-test* was used for continuous variables. Hardy–Weinberg equilibrium (HWE) of each SNP was tested by an exact test to compare the expected frequency of controls. Allele and genotype frequencies for each SNP of CC patients and control subjects were compared by χ^2^ test. Odds ratios (ORs) and 95% confidence intervals (CIs) were tested by unconditional logistic regression analysis. We used SNP analysis (http://pngu.mgh.harvard.edu/Purcell/plink/), website software to test the associations between certain SNPs and the risk of CC in five models (Codominant, Dominant, Recessive, Over-dominant and Log-additive model). For all results, *p* values presented in this study are two-sided and *p* < 0.05 was considered to represent statistically significant.

## Abbreviations

SNP: single nucleotide polymorphism
CC: cervical cancer
MAF: minor allele frequencies
HWE: Hardy-Weinberg Equilibrium
OR: odds ratio
CI: confidence interval.
